# Statins and Metformin Use Is Associated with Lower PSA Levels in Prostate Cancer Patients Presenting for Radiation Therapy

**DOI:** 10.4236/jct.2017.82007

**Published:** 2017-02-06

**Authors:** Xiaonan Liu, Jing Li, Steven E. Schild, Michael H. Schild, William Wong, Sujay Vora, Michael G. Herman, Mirek Fatyga

**Affiliations:** 1 School of Computing, Informatics, Decision Systems Engineering, Arizona State University, Tempe, AZ, USA; 2 Department of Radiation Oncology, Mayo Clinic Arizona, Phoenix, AZ, USA; 3 Department of Radiation Oncology, Mayo Clinic, Rochester, AZ, USA

**Keywords:** PSA, Statins, Metformin, Prostate Cancer

## Abstract

**Background:**

A possible association between the level of prostate specific antigen (PSA) and the use of some commonly prescribed medications has been reported in recent studies. Most of these studies were carried out in general populations of men who were screened for prostate cancer using the PSA test. We reported on the association between the initial PSA level and the use of statins, metformin and alpha-blockers in patients who were diagnosed with prostate cancer and presented for radiation therapy.

**Methods:**

Three hundred and eighty one patients treated between the years of 2000-2005 and 2009-2012 were included in this retrospective study. The information about statin, metformin and alpha-blockers use was recorded immediately prior to treatment. Differences in PSA levels prior to treatment by medication status were estimated using univa-riate and multivariate linear regression on log PSA values.

**Results:**

Compared with men who were not on these medications, the PSA level at presentation was 20% lower for statin users (p = 0.002) and 33% lower for metformin users (p = 0.004). We did not observe statistically significant associations between the use of statins or metformin and cancer stage, National Comprehensive Cancer Network (NCCN) risk score, or therapy outcome. A statistically significant association between the NCCN risk score and the use of alpha-blockers was observed (p = 0.002).

**Conclusions:**

We found that statins and metformin were associated with lower PSA levels in prostate cancer patients to an extent that could influence management decisions. We found no statistically significant associations between the use of these medications and treatment outcomes.

## 1. Introduction

Recent observational studies suggest that PSA levels can be reduced by statins [1] [2] [3] [4] and metformin [5] that are widely used to treat hypercholesterolemia and type II diabetes, respectively.

The mechanism by which statins can influence PSA levels remains unclear. A link between cholesterol and PSA has been suggested [1], but a later study which controlled for cholesterol levels did not confirm it [2]. Many other cellular mechanisms have been discussed as plausible [6] but none has been proven conclusively. Multiple observations of a negative association between statin use and PSA level, when combined with suggestions of possible biological mechanisms, have led to a hypothesis that lower PSA levels can be an indication that statins may reduce the risk of developing prostate cancer or the risk of disease progression [7]. However, subsequent studies of possible association between the use of statins and the risk of cancer or disease progression have led to inconsistent results [8]-[14].

The mechanism by which metformin can influence PSA levels remains unclear. A number of *in-vitro* studies suggested that the use of metformin could have a protective effect against prostate cancer or delay disease progression [15] [16] [17] [18]. Clinical and epidemiological studies have been inconclusive, suggesting no impact of metformin use on prostate cancer risk [19] [20] but also suggesting a possibility of a beneficial impact on disease progression and survival [19] [21].

Most studies of associations between PSA levels and the use of statins or metformin were retrospective and performed on general populations of men who were being screened for prostate cancer. Studies done on general populations offer the advantage of higher numbers but also introduce many possible confounding factors. Furthermore, if a negative association between PSA levels and use of medications in the general population was caused primarily by the reduction in risk of developing the disease, the same effect could be significantly smaller or even entirely absent in the population of patients who were already diagnosed with cancer. As an example, a study of PSA levels in a population of diagnosed prostate cancer patients observed a fairly strong negative association between PSA levels and the use of aspirin, but no significant association with the use of statins [22].

Inaccurate determination of PSA level prior to and after treatment could significantly influence management decisions. PSA level is one of the factors used to assign prostate cancer patients to a risk group, and it is also a sole factor which is used to detect treatment failure in patients who are not yet clinically symptomatic (biochemical failure) [23].

The purpose of the present study was to both verify and quantify the effects that statins and metformin may have on PSA levels in the population of prostate cancer patients who presented for radiation therapy. By studying a population of men who were already diagnosed with cancer, we could also take advantage of the diagnosis and follow-up information to ask whether there was any evidence that observed associations between the PSA level and the use of medications influenced management decisions to an extent that would affect treatment outcomes.

## 2. Materials and Methods

### 2.1. Patients

We combined two IRB approved studies of patients who were treated between the years of 2000-2005 (302 patients) [24] and 2009-2012 (79 patients) [25]. Both groups were treated with Intensity Modulated Radiation Therapy (IMRT), with the second group receiving an additional boost to the region of prostate with the greatest disease burden as identified by MRI studies. Patient characteristics are shown in **Table 1**. The number of patients in each group was determined by the requirements of each respective protocol and was not optimized for the present, retrospective study.

The use of four medications was recorded in the database: statins, metformin, alpha-blockers and androgen deprivation therapy (ADT). The use of these medications was recorded only at baseline and no dosage or duration of use information was recorded.

PSA levels were recorded prior to treatment, 4 months after treatment, and subsequently monitored in 6 - 12 months intervals.

### 2.2. Statistical Methods

In univariate analysis, a simple regression was used to model the association between the PSA level and the use of each medication individually. A logarithm transformation of the PSA level was performed prior to the regression modeling in order to account for the heavy tail distribution of the PSA level. The p-values of regression coefficients were computed using a t-test.

In multivariate analysis, a multiple regression was used to associate a logarithm transformation of the PSA level with statins, metformin, alpha-blockers, prostate volume, age, ADT and two-way interactions between these predictors. A stepwise procedure was used to select the significant predictors which were included in the final model.

To model the association between the diagnosis (staging) and the use of a medication, a chi-square test was used if the diagnosis (staging) variable was categorical and a t test is used if it was continuous.

To model the association between a clinical outcome and the use of a medication, a survival analysis was performed that used a Kaplan-Meier estimator to estimate the survival fraction of each of the two strata (*i.e.,* use and not use of the medication), and a log-rank test for assessing if there was a statistically significant difference between the survival fractions of the two strata.

Patients in the first group of 302 patients were followed after treatment for up to eleven years (median of 91 months, range 6 - 138 months) while patients in the second group of 79 patients were followed for up to 4.5 years (median of 26 months, range 3 - 54 months). The majority of the analysis presented in this work was not sensitive to the follow up period, with an exception of biochemical and clinical failure. We combined both databases to maximize the size of the sample for the majority of the analysis, but analyzed biochemical and clinical failure using combined databases and the database with longer follow up period to test for a possible bias which could be introduced by the difference in follow up periods.

Statistical package “R” [26] was used in all the analysis presented in the paper.

## 3. Results

A summary of patient characteristics is presented in **Table 1** with stratifications for medication use and the treatment protocol. The first two columns summarize characteristics of patients in two protocols that contributed patients to the present study [24] [25]. The third column summarizes characteristics of all patients combined, as they were used in the data analysis for the present study. The remaining three right-most columns summarize patient characteristics for both groups combined but after stratification for the use of medications. One generally observes that patient characteristics do not vary significantly among the groups.

Data used in statistical analysis of PSA levels is summarized in **Table 2**, showing mean and standard deviation of the PSA distribution for groups of patients who were stratified by the use of medications and their diabetes status. The two columns on the right side of the table include mean PSA levels after stratification into patients who did and did not receive an ADT therapy. Mean PSA levels in patients receiving ADT therapy are elevated due to a selection bias because patients who presented with higher PSA levels were more likely to be offered the ADT therapy. One notes that a bias towards lower PSA levels in users of statins and metformin is quite apparent in the raw data regardless of the ADT status.

Results of univariate analysis including one medication at a time are shown in **Table 3**. Only the use of statins and metformin is included because alpha blockers showed no statistically significant association with PSA in univariate analysis. Two additional columns show results of univariate analysis after stratification for ADT therapy. The use of Statins and Metformin appears to be associated with lower PSA values, which agrees with trends that can be seen in **Table 2**. The correlations are statistically significant, though the significance becomes marginal after stratification for ADT therapy, most likely because the number of patients in each of the two groups becomes too low.

Results of multivariate analyses which included statins, metformin, alpha-blockers, age, prostate volume, and ADT are shown in **Table 4**. Only significant results are shown. The upper portion of the table summarizes results of the analysis without interaction terms, while the lower portion summarizes results of the analysis with interaction terms. The results shown in **Table 4** can be summarized as follows: compared with men who were not on medication, the PSA level at presentation was 15% lower for statin users (*p* = 0.03) and 29% lower for metformin users (*p* < 0.02). When prostate volume increased by 1 ml, the PSA level increased by 0.3% (*p* < 0.01). A very strong association between ADT and PSA was a result of selection bias because the decision to recommend ADT is based on the risk group which is correlated the PSA level. A multivariate analysis with correlation terms revealed one possible correlation between the use of statins and the use of metformin, but the statistical significance of this correlation was marginal. If the interaction terms are included: compared to patients who did not use statins or metformin, users of statins alone had PSA levels that were 18% lower, users of metformin alone had PSA levels that were lower by 47%, and patients who used both statins and metformin had PSA levels that were lower by 24%.

We compared PSA distributions recorded in patients who took metformin with PSA distributions in patients who were diabetic but did not take metformin (**Table 2**), using Wilcoxon and Kolmogorov-Smirnov tests. Results of both tests show that PSA distributions in these two groups of patients were significantly different (*p* = 0.03).

We searched for associations between the diagnosis (staging) and the use of medications. We found only one statistically significant association between the use of alpha-blockers and the overall National Comprehensive Cancer Network (NCCN) score [27], suggesting that patients who use alpha-blockers may have a higher NCCN risk score. Results are summarized in **Table 5** and show that the p-values for all tests are high, except for the correlation between the NCCN risk scores and the use of alpha-blockers which is highly significant.

**Figure 1:**
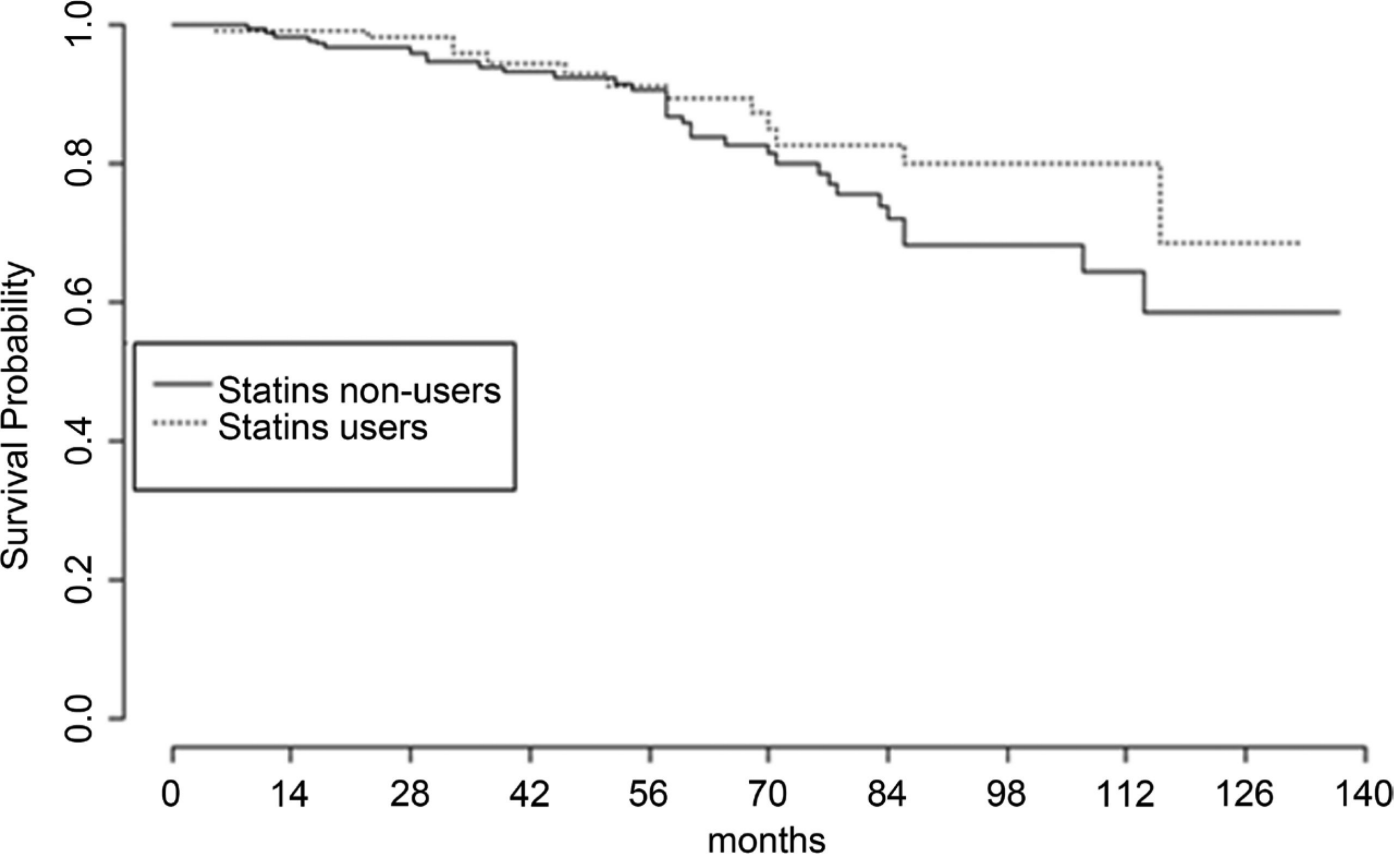
Kaplan-Meier analysis of biochemical failure. The difference between two groups is not statistically significant (*p* = 0.38).

We searched for associations between clinical outcomes and the use of medications. We used Kaplan-Meier analysis to search for associations with overall survival, disease-specific mortality, local failure, distant failure and biochemical failure. No statistically significant associations were found (**Table 6**). However, the data suggests a possible association between the use of statins and biochemical failure. Results of survival analysis with biochemical failure as the endpoint are shown in **Figure 1** While the numeric data is suggestive, the difference between statin users and non-users is not statistically significant (p = 0.38).

No significant difference was identified in the results of a similar search for associations between clinical outcomes and the use of medications which excluded the 79 patients who had a shorter follow up time.

## 4. Discussion

Our analysis revealed that the use of statins and metformin were associated with lower PSA levels in patients who were diagnosed with prostate cancer and presented for radiation therapy. The effect was identified in both univariate and multivariate analyses including possible interactions between medications and other factors that could affect the PSA level.

We performed univariate analysis for all patients in the database and following stratification into two subgroups based on the ADT therapy. Lower PSA levels in patients who took statins or metformin were observed for all three analyses although the statistical significance was marginal for some of the results after stratification, most likely due to a lower number of patients in each group.

Results of multivariate analysis also showed that patients who used statins and metformin had lower PSA levels. Results suggested a possibility of interactions between statins and metformin but the interaction term had marginal statistical significance. Interactions between medications were reported in at least one prior study [2], suggesting that the results of retrospective studies should be interpreted with caution due to possible confounding factors.

Multiple studies examining the association between PSA levels and the use of statins reported negative associations which ranged from −4.6% [5] to −40% [3], with several studies reporting values in the middle of this range [1] [2] [4]. Results of our study fell in the middle of the range which was reported in the literature. Our results did not agree with at least one retrospective study in the population of cancer patients by Algotar *et al.* [22] as their study found a strong negative association between the use of aspirin and PSA levels, but not between the use of statins and PSA levels. The study by Algotar *et al.* was based on a population of 140 patients with confirmed diagnosis of prostate cancer, who agreed to forego active treatment and were enrolled in the Selenium supplementation trial. The disagreement between the two studies could have been caused by a smaller sample size in the Algotar study, but it is also possible that the differences arose due to unidentified interactions between medications that the patients may have been taking. For example, the analysis in the Algotar study was not corrected for possible effects of Selenium supplementation. This disagreement underscores a need for carefully controlled prospective studies to confirm the effect that we report. An important limitation of studies on populations of cancer patients, when compared to studies on general populations, is significantly lower sample size, particularly in a single institution setting. A meta-analysis of multiple studies may be needed to fully understand patterns of associations between the use of medications and PSA levels.

A recent Swedish study of 185,667 men undergoing PSA screening reported 14% lower PSA levels at a first screening test in men who used metformin compared to men who did not [5]. Results of our study suggest an effect which is twice as large, but both studies may be consistent due to wider error intervals in our study. Since the same study showed a negative association between the use of Insulin and PSA levels (−16%) we analyzed our data to determine if the lower PSA levels could be caused by diabetes alone. We compared men who used metformin to men who were diabetic but did not use metformin (**Table 2**) and determined that both groups had significantly different PSA distributions (p = 0.03) and there was no indication that patients who were diabetic but did not take metformin had lower PSA levels than those who were not diabetic.

We did not observe an association between the use of statins or metformin and the stage of prostate cancer (**Table 5**) which suggests that men who use sta-tins and metformin have lower PSA levels but are not more likely to have a less (or more) advanced malignancy.

We did not observe an association between the use of statins or metformin and the NCCN risk group that patients were assigned to (**Table 5**). This lack of association implies that any bias in an assignment to a risk group that might have been caused by lower PSA levels was not strong enough to create a statistically significant signal in a population of 381 patients.

We did not observe statistically significant associations between the use of medications and local failure, distant failure or biochemical failure (**Table 6**). Nonetheless the Kaplan-Meier analysis hints at a possible association between the use of statins and the likelihood of biochemical failure (**Figure 1**) that did not reach statistical significance. Larger and preferably prospective study would be needed to determine if the effect is real and whether it indicates a delay in a detection of the biochemical failure or a genuine reduction in a risk of disease progression.

Metformin has been previously studied *in vitro* as a possible agent that could have protective effect against prostate cancer or delay disease progression [15] [16] [17] [18]. Clinical and epidemiological studies have been inconclusive, suggesting no impact of metformin use on prostate cancer risk [19] [20] but also suggesting a possibility of a beneficial impact on disease progression and survival. [21] [19]. Studies that observed lower PSA levels in general populations of metformin users [5] could be interpreted as an indication of a reduced risk of prostate cancer in these populations. Results of our study suggest that a similar negative association between PSA levels and the use of metformin can also be seen in the population of patients with a confirmed diagnosis of prostate cancer. This finding suggests that the use of metformin may be associated with lower PSA levels for as yet undetermined reasons, but lower PSA levels alone are not necessarily an indication of a lower risk of developing prostate cancer.

Numerous biological mechanism that could lower PSA levels in patients who use statins have been suggested [1] [6]. Multiple observations of negative associations between statin use and PSA levels, when combined with suggestions of possible biological mechanisms, have led to a hypothesis that lower PSA levels can be an indication that statins may reduce the risk of developing prostate cancer or the risk of disease progression [8]. However, subsequent studies of possible associations between the use of statins and the risk of cancer or disease progression have led to inconsistent results [8]-[14]. Results of our study confirmed that the negative association between PSA levels and the use of statins can also be seen in patients who were already diagnosed with prostate cancer. This finding suggests that the use of statins is associated with lower PSA levels, but lower PSA levels alone are not necessarily an indication of a lower risk of developing prostate cancer.

Results of the present study suggest that the negative association between PSA levels and the use of statins or metformin did not significantly impact the clinical management of prostate cancer patients. We did not observe an association between the use of these medications and an assignment of patients to a risk group, or an association between the use of medications and clinical endpoints. One should note, however, that the number of patients in our study may have been too small to establish statistically significant correlations between the use of medications and clinical endpoints. We were able to determine that the use of statins and metformin was associated with lower PSA levels, but larger studies may be needed to determine whether these associations have an impact on the clinical practice. A 2011 study by Kollmeier *et al.* [28] reported a statistically significant improvement in biochemical control for patients who used statins, were diagnosed with high risk prostate cancer according to NCCN criteria, and were treated with radiation therapy. This study included a total of 1711 patients of whom 489 were classified as high risk patients. No significant association between the use of statins and distant metastasis free survival was found however, which raises a question whether observed improvements in biochemical control were caused by a delay in detecting a rise in PSA levels or a genuine improvement in relapse-free survival. A large and more detailed study of PSA kinetics would most likely be needed to distinguish between these two possibilities.

## 5. Limitations of This Study

The retrospective nature of the study could have introduced uncontrolled biases. The use of only four medications was recorded and no dosage or duration of use information was recorded. A larger, prospective study is recommended to verify our findings.

## 6. Conclusion

The use of statins and metformin was associated with lower PSA levels in prostate cancer patients to an extent that could potentially affect management decisions and a detection of biochemical failure. No statistically significant association between the use of statins or metformin and clinical outcomes of radiation therapy for prostate cancer was observed.

## Figures and Tables

**Table 1:** Summary of patient characteristics.

	Patients treated 2000-2005	Patients treated 2009-2012	All patients	Statins users	Metformin users	Statins and Metformin users
Number of patients	302	79	381	146	27	12
Age [years]	74.3 ± 5.6	74.9 ± 7	74.4 ± 6.0	74.4 ± 6.0	72.2 ± 6.9	71.0 ± 8.3
Follow up time (median) [months]	91 [6 - 138]	26 [3 - 54]	70 [3 - 138]	69.8 [3 - 136]	62 [3 - 129]	45 [3 - 128]
Prostate Volume (mean) [cc]	78.9 ± 32.0	75.3 ± 26.6	78.0 ± 30.7	78.0 ± 30.8	91.4 ± 32	77.0 ± 14
Baseline PSA (mean)	9.1 ± 8.0	8.6 ± 6.7	9.0 ± 7.0	7.0 ± 4.8	5.4 ± 2.1	5.3 ± 1.6
Baseline PSA (median)	7.0	6.7	6.8	6.1	5.3	5.2
Diabetes	11% (N = 33)	14% (N = 14)	11.7% (N = 47)	13% (N = 19)	100% (N = 27)	100% (N = 12)
Hormonal Therapy [%]	35% (N = 107)	42% (N = 46)	40% (N = 153)	33% (N = 98)	32% (N = 9)	8.3% (N = 1)
Gleason > 6 [%]	56% (N = 169)	67% (N = 53)	58% (N = 222)	56% (N = 82)	37% (N = 10)	67% (N = 8)
Gleason = 8 - 10 [%]	17.9% (N = 54)	16.5% (N = 13)	18% (N = 67)	16% (N = 23)	15% (N = 4)	0% (N = 0)
T stage > T2a [%]	25.8% (N = 78)	43% (N = 34)	29% (N = 112)	27% (N = 39)	18.5% (N = 5)	0% (N = 0)
Biochemical Failure	19.7% (N = 59)	7.6% (N = 6)	17% (N = 65)	14% (N = 20)	15.4% (N = 4)	0% (N = 0)
Local Failure	3.3% (N = 10)	2.5% (N = 2)	3% (N = 12)	3.5% (N = 5)	3.7% (N = 1)	0% (N = 0)
Distant Failure	5.7% (N = 17)	5.1% (N = 4)	5.5% (N = 21)	3.5% (N = 5)	3.7% (N = 1)	0% (N = 0)
Died of Prostate Cancer	4.3% (N = 13)	1% (N = 1)	3.5% (N = 14)	2.7% (N = 4)	0% (N = 0)	0% (N = 0)
Died of Any Cause	24% (N = 72)	7.5% (N = 6)	21% (N = 79)	16% (N = 24)	19% (N = 5)	8% (N = 1)
Treatment Technique	5 field IMRT	7 field IMRT				
Dose Prescription	Median Dose 75.6Gy in 1.8 Gy fractions	Median dose 80.3 Gy in 1.8 Gy fractions				

**Table 2:** Means and Standard Deviations of PSA distributions for groups of patients stratified by medication use and ADT therapy.

	All patients	Patients receiving ADT therapy	Patients not receiving ADT therapy
Statins users	7.0 ± 4.8 (N = 146)	9.6 ± 6.9 (N = 48)	6.2 ± 3.5 (N = 98)
Statins non-users	10.1 ± 9 (N = 255)	13.8 ± 11.9 (N = 101)	7.5 ± 4.2 (N = 154)
Metformin users	5.4 ± 2.1 (N = 27)	6.2 ± 1.4 (N=9)	5.1 ± 2.3 (N = 18)
Metformin non-users	9.2 ± 7.9 (N = 373)	12.7 ± 10.9 (N = 140)	7.2 ± 4.1 (N = 233)
Metformin non-users who have diabetes	13.6 ± 15.3 (N = 21)	22.7 ± 19.8 (N = 8)	7.8 ± 8.8 (N = 13)
Metformin and Statin users	5.3 ± 1.6 (N = 12)	7.3 ± undef (N = 1)	5.2 ± 1.5 (N = 11)
Alpha-blockers users	7.5 ± 4.7 (N = 98)	8.2 ± 4.7 (N = 38)	7.0 ± 4.7 (N = 60)
Alpha-blockers non-users	9.5 ± 8.4 (N = 302)	13.8 ± 11.8 (N = 111)	7.0 ± 3.9 (N = 191)

**Table 3:** Results of univariate regression analysis for all patients and after stratification for ADT.

	All patients	Patients on ADT	Patients not on ADT
Statins	−19.8% [−30.3%, −8.2%] (p = 0.002	−26% [−42.0%, −4.7%] (p = 0.02)	−14% [−26.2%, +0.7%] (p = 0.06)
Metformin	−33.0% [−48.1%, −12.8%] (p = 0.004)	−36% [−61.6%, +5.2%] (p = 0.08)	−24% [−43.4%, +3.8%] (p = 0.09)

**Table 4:** Results of multivariate analysis including statins, metformin, alpha-blockers, age, prostate volume, ADT. Only significant results are shown after stepwise procedure to select significant predictors. A very strong positive association between hormone use and PSA level is caused by a selection bias (patients with high PSA levels are preferentially prescribed hormone treatment). 95%CL limits are shown in parenthesis below.

Multivariate with no interactions					
	Statins	Metformin	Prostate volume	ADT	Statins*Metformin
Coefficient	−0.16 [−0.23, −0.09]	−0.34 [−0.48, −0.2]	0.003 [0.002, 0.004]	0.46 [0.39, 0.53]	N/A
*p-value*	0.03	<0.02	<0.02	<0.001	N/A
Multivariate with interactions					
Coefficient	−0.2 [−0.28, −0.12]	−0.64 [−0.84, −0.44]	0.003 [0.002, 0.004]	0.46 [0.39, 0.53]	0.56 [0.27, 0.83]
*p-value*	<0.01	<0.01	<0.01	<0.001	0.05

**Table 5:** Tests of possible associations between initial diagnosis and use of medications.

	Method	*p-value*
Statins versus T-stage	chi-square test	0.29
Statins versus Gleason Score	t test	0.26
Statins versus NCCN risk score	chi-square test	0.83
Metformin versus T-stage	chi-square test	0.38
Metformin versus Gleason score	t test	0.31
Metformin versus NCCN risk score	chi-square test	0.55
Alpha-blockers versus T-stage	chi-square test	0.62
Alpha-blockers versus Gleason score	t test	0.9
Alpha-blockers versus NCCN risk score	chi-square test	0.002

**Table 6:** results of Kaplan-Meier analysis of possible associations between the use of medications and clinical endpoints: biochemical failure, local failure, distant failure, disease specific survival and overall survival. No statistically significant associations were found.

Endpoint	Log Rank	Wilcoxon
Biochemical Failure Statins	0.38	0.75
Biochemical Failure Metformin	0.6	0.88
Local Failure Statins	0.62	0.94
Local Failure Metformin	0.72	0.9
Distant Failure Statins	0.4	0.58
Distant Failure Metformin	0.94	0.36
Disease Specific Survival Statins	0.63	0.9
Disease Specific Survival Metformin	0.33	0.37
Overall Survival Statins	0.3	0.8
Overall Survival Metformin	0.99	0.73
